# Bifunctional supramolecular nanofiber inhibits atherosclerosis by enhancing plaque stability and anti-inflammation in apoE^-/-^ mice

**DOI:** 10.7150/thno.48410

**Published:** 2020-08-13

**Authors:** Yuna Shang, Chuanrui Ma, Jing Zhang, Zhongyan Wang, Chunhua Ren, Xin Luo, Rong Peng, Jingfei Liu, Jingyuan Mao, Yang Shi, Guanwei Fan

**Affiliations:** 1 State Key Laboratory of Medicinal Chemical Biology, Key Laboratory of Bioactive Materials, Ministry of Education, College of Life Sciences, Nankai University, Tianjin 300071, P. R. China.; 2 First Teaching Hospital of Tianjin University of Traditional Chinese Medicine, Tianjin Key Laboratory of Translational Research of TCM Prescription and Syndrome, Tianjin 300381, P. R. China.; 3 State Key Laboratory of Modern Chinese Medicine, Tianjin University of Traditional Chinese Medicine, Tianjin 300193, P. R. China.; 4 Tianjin Key Laboratory of Radiation Medicine and Molecular Nuclear Medicine, Institute of Radiation Medicine, Chinese Academy of Medical Sciences & Peking Union Medical College, Tianjin 300192, P. R. China.

**Keywords:** IGF-1 mimetic peptide, bifunctional supramolecular nanofiber, cholesterol efflux, foam cells, atherosclerosis, inflammation

## Abstract

**Background and Purpose:** Atherosclerosis is vascular disease of chronic inflammation and lipid disorder, which is a major cause of coronary heart disease. Foam cell formation is key progress during the atherosclerosis development. Insulin-like growth factor (IGF)-1 is a growth hormone that plays a crucial role in growth, metabolism, and homeostasis. Previous studies have demonstrated that increase in circulating IGF-1 can reduce atherosclerotic burden. However, active IGF-1 is characterized with poor tissue retention and is at a very low level in circulation system. Therefore, supplementation of exogenous IGF-1 to restore the physiological level is a promising approach to inhibit atherosclerosis. In this study, we develop a self-assembling, anti-inflammatory drug-modified peptide derived from IGF-1 to mimic IGF-1 bioactivity and simultaneously with an anti-inflammatory property for the treatment of atherosclerosis.

**Methods:** ApoE^-/-^ mice were subcutaneously (s.c.) injected with the different hydrogels or natural IGF-1 protein solution per week and simultaneously fed a high-fat diet for 16 weeks. Atherosclerotic lesion formation and stability were assessed after treatment. Moreover, peritoneal macrophage and serum samples were collected to determine lipid profile and inflammatory cytokines. Concurrently, we determined the effect of bifunctional supramolecular nanofibers/hydrogel on cholesterol efflux, foam cell formation, phenotypic transformation of VSMC to macrophage-like cells, and macrophage polarization *in vitro* or *in vivo*.

**Results:** Bifunctional supramolecular nanofibers/hydrogel for the treatment of atherosclerosis was formed by a short peptide consisting of a tetrapeptide SSSR from C-region of growth factor IGF-1, an anti-inflammatory drug naproxen (Npx), and a powerful self-assembling D-peptide ^D^F^D^F. The resulting hydrogel of Npx-^D^F^D^FGSSSR (Hydrogel 1, H1) possessed both the anti-inflammatory and IGF-1 mimicking properties, and it efficiently promoted the expression of ABCA1 and ABCG1, thereby significantly reducing cholesterol accumulation in macrophages and preventing foam cell formation. Moreover, H1 markedly inhibited the transformation of vascular smooth muscle cells (VSMCs) into macrophage-like cells which also contributed to foam cell formation. In addition, H1 significantly reduced the inflammatory response *in vitro* and *in vivo*. Most importantly, the IGF-1 mimetic peptide showed comparable performance to IGF-1 i*n vivo* and inhibited atherosclerosis by markedly reducing lesion area and enhancing plaque stability.

**Conclusions:** Our study provides a novel supramolecular nanomaterial to inhibit pathological progress of atherosclerosis through regulating cholesterol efflux and inflammation, which may contribute to the development of a promising nanomedicine for the treatment of atherosclerosis in the clinic.

## Introduction

Atherosclerosis is a major health problem worldwide with significant morbidity and mortality 1. The development of therapeutics and approaches to efficiently treat atherosclerosis is very important for human health. Though a complete understanding of the pathogenesis of atherosclerosis remains elusive, the oxidative stress and inflammation play important roles in the occurrence and progression of atherosclerosis 2. Inflammation-driven endothelial dysfunction initiates and contributes to the progression of atherosclerosis. The dysfunctional endothelium develops to a state that is conducive to inflammatory cell adhesion, rolling, and migration in the subendothelial region, where monocytes differentiate into macrophages and subsequently transform into lipid-filled foam cells 3, 4. Accumulation of excess cholesterol within the arteries further promotes endothelium dysfunction, which leads to increased production of proinflammatory cytokines and reactive oxygen species, overexpression of adhesion molecules, and in turn, acceleration of cholesterol deposition and amplification of inflammation 5. Therefore, vascular inflammation and lipid disorder are linked in a vicious cycle. As such, efficient inhibition of inflammatory response during the pathological process of atherosclerosis may be a promising approach to treat atherosclerosis.

Insulin-like growth factor (IGF)-1 exerts both anti-inflammatory and anti-oxidant properties, and it is a growth hormone that plays a crucial role in human development, growth, metabolism, and homeostasis 6. The IGF-1 deficiency has been linked to increased atherosclerotic complications in most observational studies 7. In a prospective study on the effect of IGF-1 levels, low circulating IGF-I levels were associated with increased risk of ischemic heart disease during a 15-year follow-up period 8. Conversely, high free IGF-1 was related to decrease carotid plaques and coronary artery disease in participants of the Rotterdam Elderly Study 9. Noticeably, in clinic, recombinant IGF-1 replacement in men reduces levels of serum lipoprotein concentrations which is the major contributor to atherosclerosis development 10, 11. Moreover, in an atherosclerotic animal model, low levels of IGF-1 in circulation are associated with more atherogenesis 12 and *vice versa*; further, an increase in circulating IGF-1 reduces atherosclerotic burden 13. However, less than 1% of total IGF-1 is present as its free form 14. IGF-1 receptor is a receptor tyrosine kinase that regulates cell growth and proliferation, and can be activated by IGF1, IGF2, and insulin 15. Of note, only free IGF-1 is believed to be active as it can readily cross through the endothelium and interact with its own receptor. Therefore, supplementation of exogenous IGF-1 to restore the physiological levels of IGF-1 is a very promising approach to exert antiatherogenic function.

Supplementation of exogenous IGF-1 can effectively inhibit atherosclerosis 16. However, natural or recombinant human IGF-1 is expensive, and prone to inactivation by endogenous digestion enzymes 17. Therefore, it is urgent to develop an IGF-1 replacement that is simple to synthesize and prepare and simultaneously has biological activity comparable to that of natural proteins. Given the antiatherogenic effects of IGF-1 and beneficial effect of anti-inflammation strategy on atherosclerosis development, we developed an IGF-1-derived peptide modified by an anti-inflammatory drug, which has the characteristics of IGF-1 bioactivity, self-assembling, and enhanced anti-inflammatory property. Self-assembling peptides have been widely used in drug delivery 18-22, biological analysis 23-28, tissue engineering 24, 26, 29-32 , and immune adjuvants 33-35 due to advantages of peptides such as ease of design and preparation 36-38, good biocompatibility 39-43, and excellent bioactivity 44-46. It has been widely reported that bioactive peptides can be assembled into nanofibers by covalent modification with self-assembling peptides, thereby improving their stability, tissue retention and bioavailability to boost their biological functions. We recently reported a self-assembling peptide by covalently attaching a peptide with 12 amino acids from IGF-1 to a β-sheet forming peptide Nap-FF, and the resulting nanofibers show superior bioactivity to native protein IGF-1 17, thus effectively improving lower limb ischemia. In this study, we introduce a novel bifunctional supramolecular nanofiber with both IGF-1 bioactivity and anti-inflammatory property to efficiently inhibit atherosclerosis.

## Results

### Molecular design and preparation of supramolecular nanofibers

**Figure 1:**
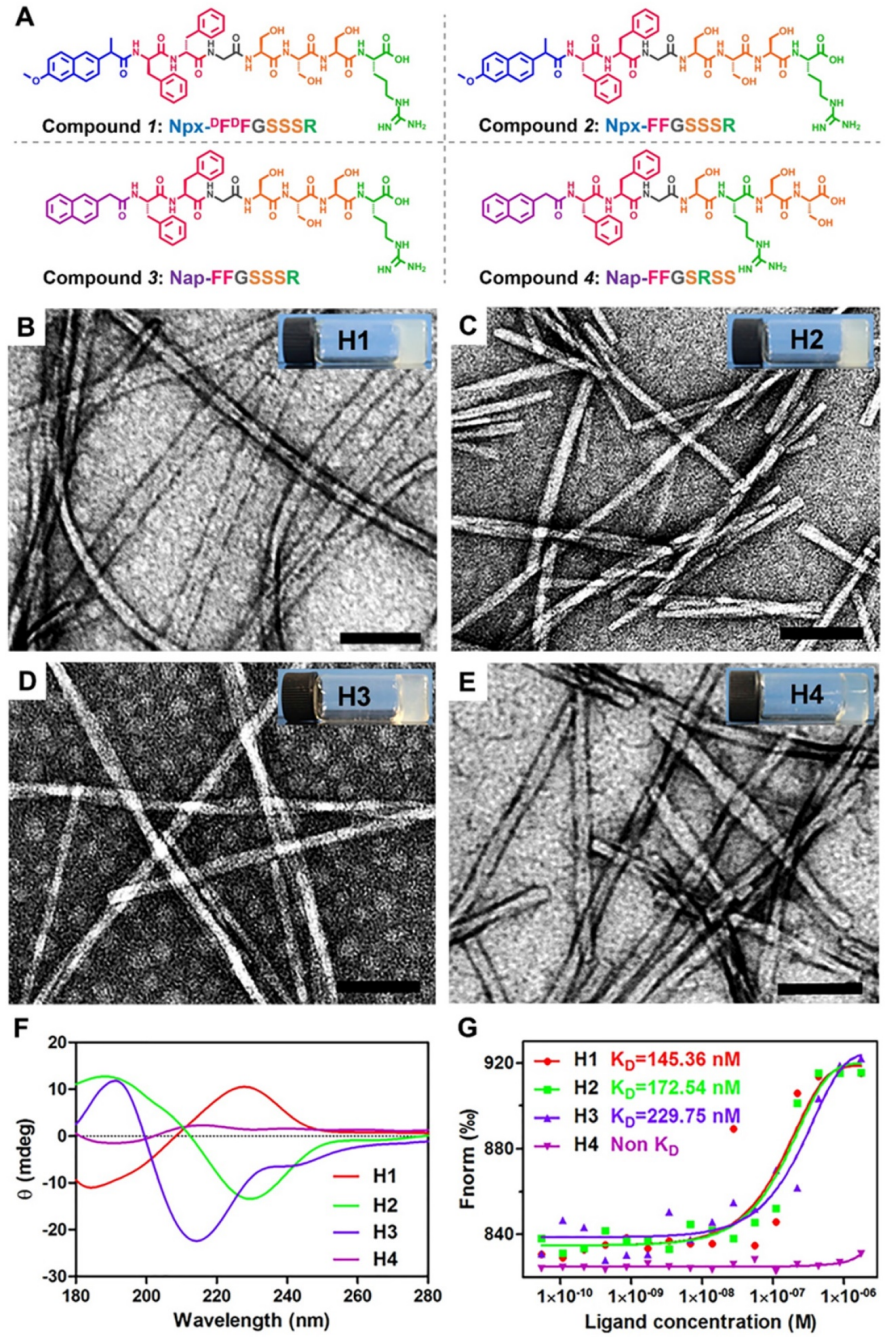
** Preparation and characterization of supramolecular nanofibers. (A)** Chemical structure of compounds ***1***-***4***. **(B-E)** TEM images and optical images of the hydrogels 1-4 in PBS buffer solution at the concentration of 0.5 wt%. **(F)** Circular dichroism spectra of hydrogels 1-4 (0.5 wt %).** (G)** The binding constants of hydrogels 1-4 with rhIGF-1R. The K_D_ values was labeled in the corresponding curves.

Natural and recombinant human IGF-1 proteins (rhIGF-1) are limited in short half-lifes in biological systems and poor tissue retention. Hence, it is difficult to maintain the bioactivity of IGF-1 protein during storage, transportation, and incorporation into biomaterials. The C domain of GYGSSSRRAPQT in IGF-1 regulates its receptor binding and directly affects the biological activity of IGF-1. Nishida *et al.* reported that the C-domain of IGF-1 can promote the healing of corneal epithelial wounds. They screened a series of 12-amino acids of GST fusion protein and obtained mutants whose various amino acids were replaced by alanine. Their results indicated that the replacement of any amino acid in the tetrapeptide sequence SSSR led to a loss of activity. Therefore, the tetrapeptide SSSR was crucial for the biological activity of IGF-1. We recently reported a self-assembling peptide Nap-FFGGYGSSSRRAPQT with a high binding affinity to the receptor of IGF-1, and therefore it possessed a similar bioactivity to IGF-1. In addition, pioneering works indicated that self-assembling peptides of D-amino acids exhibited better stabilities both *in vitro* and *in vivo* than their L-counterparts. More importantly, an inflammatory response and an excessive lipid accumulation within arteries are two major contributors to atherogenesis, and previous studies have shown that injection of IGF-1 can effectively inhibit the development of atherosclerosis in an animal model 13. Moreover, naproxen (Npx) is an anti-inflammatory drug, which is commonly used to treat chronic inflammation 47. Based on the information, we designed and synthesized compound ***1*** (Npx-^D^F^D^FGSSSR, Figure 1A) containing ^D^F^D^F and the tetrapeptide SSSR from IGF-1 with the anti-inflammatory drug naproxen (Npx) as a capping group to explore its therapeutic effect on atherosclerosis. To further explore the functions of each part, we also designed three control peptides: Npx-FFGSSSR (L-peptide, compound ***2***), Nap-FFGSSSR (without anti-inflammatory drug naproxen, compound ***3***) and Nap-FFGSRSS (without anti-inflammatory drug naproxen and with a scrambled tetrapeptide of SSSR, compound ***4***). The obtained peptides were characterized by ^1^H-NMR and HR-MS techniques. The peaks from 7 to 7.5 ppm could be assigned to the aromatic rings of phenylalanine and naphthalene nucleus, and the peaks near 8 ppm were the characteristic signals of amide bond in peptide chain (Figure S1). These results, in combine with the characteristic molecular weight of 999.4559 in HR-MS spectrum (Figure S2) indicated that the compound Npx-^D^F^D^FGSSSR was successfully synthesized. Similarly, characteristic peaks in ^1^H-NMR spectrum (Figure S3, Figure S5 and Figure S7) and characteristic molecular weight in mass spectrum (Figure S4, Figure S6 and Figure S8) suggested that the compounds ***2***-***4*** was also successfully synthesized, respectively.

Compounds ***1***-***4*** could self-assemble into hydrogels by the heating-cooling process in PBS buffer (pH 7.4) at a concentration of 0.5 wt% (H1-H4, respectively in Figures 1B-E), respectively. We found that four hydrogels differ in transparency. The transparency of the prepared hydrogel in the visible light range of 380 nm-780 nm is detected by UV-Vis spectroscopy (Lambda 35 UV/Vis Spectrometer, Perkin-Elmer). As shown in Figure S9, the transparency of four hydrogels increases monotonically with the increasing of the wavelength. The hydrogels 1-3 with the same tetrapeptide sequence SSSR have similar transparency and are all less than 40%, which are 20.54%, 29.11% and 35.44% respectively. Once the tetrapeptide sequence is disturbed, the transparency of the hydrogel can be significantly changed. For instance, the mean transmittance of hydrogel 4 is 71.08%. It turns out that the different arrangement of the same amino acid is very important for its gelatinous property. Transmission electron microscopy (TEM) images revealed entangled nanofibers with diameters of approximately 26.8, 14.6, 9.8, and 17.3 nm in H1-H4, respectively (Figures 1B-E). The circular dichroism (CD) spectra indicated that the peptide in H2 adopted a β-sheet conformation with a positive peak at approximately 188 nm and a negative peak at approximately 229 nm (Figure 1F). The D-peptide in H1 also exhibited a β-sheet conformation, indicating by a mirror image in the CD spectrum to that of H2 (Figure 1F). The peptide in H3 also adopted a β-sheet conformation, but the peptide in H4 had no ordered conformations at the same concentration of 0.5 wt%, as indicated by the very weak CD signals in the spectrum (Figure 1F). Previous studies showed that the β-sheet conformation was important for simulating binding of IGF-1 to its receptor 17. Therefore, we conducted microscale thermophoresis experiments to determine their binding affinity to recombinant human Insulin-like Growth Factor-I Receptor (rhIGF-1R). The results showed that the dissociated constant (K_D_) between Compounds ***1-3*** in H1-H3 and rhIGF-1R was 145.36, 172.54 and 229.75 nM respectively, while Compound ***4*** in H4 showed no binding at all. Thus, the tetrapeptide sequence SSSR was crucial for the binding to rhIGF-1R and probably the bioactivity of IGF-1.

### The IGF-1 mimetic peptide retards the development of atherosclerosis* in vivo*

**Figure 2:**
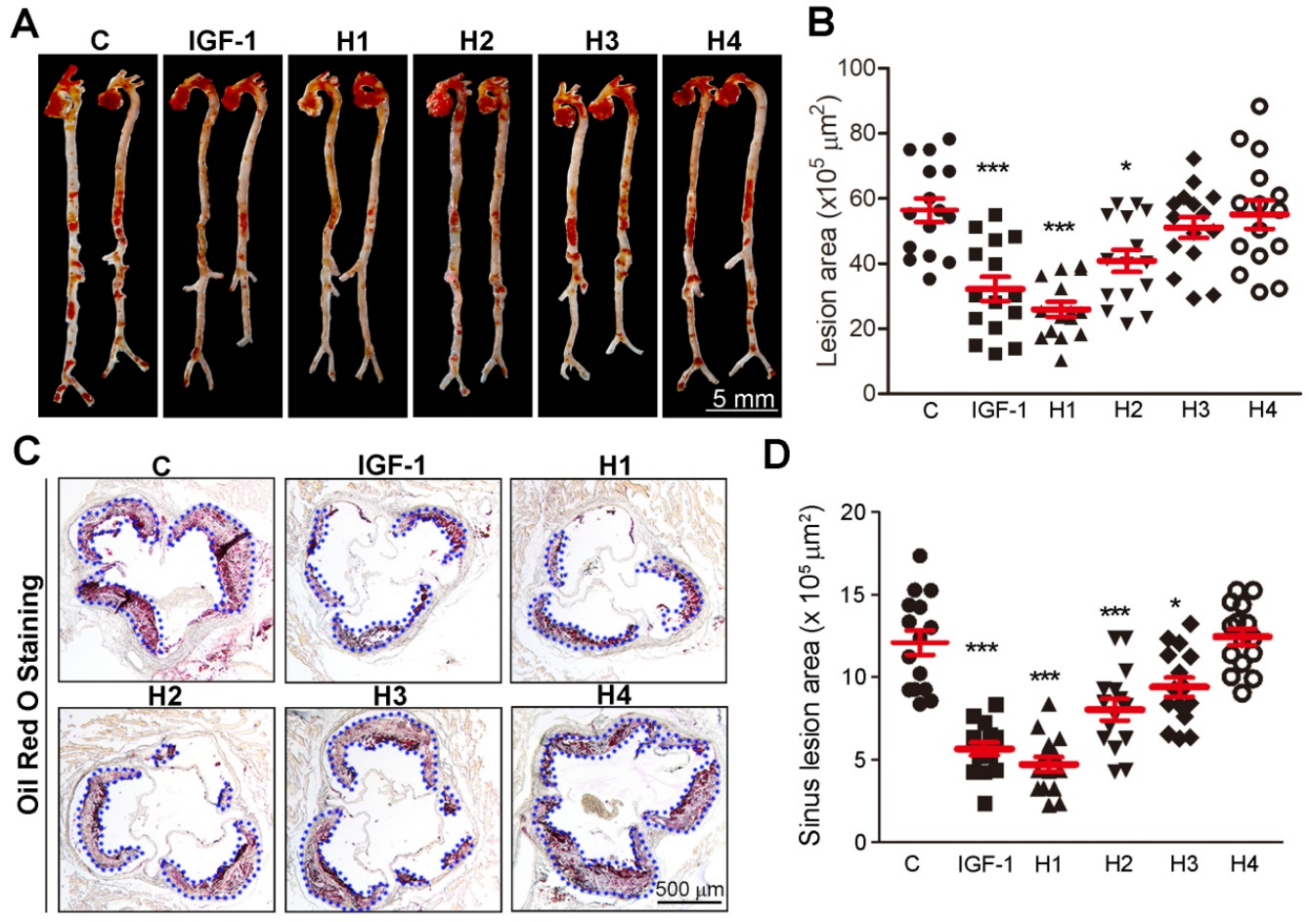
** Analysis of atherosclerotic lesions area in *en face* aortas and aortic root cross sections.** (**A-B**) Representative photomicrographs of lesions in *en face* aortas were determined by Oil Red O staining and quantified by a computer-assisted image analysis protocol, n=15. (**C-D**) Lesions in aortic root cross sections were determined by Oil Red O staining and quantified. Lesion areas were expressed as µm^2^, n = 15. Data are presented as mean ± SEM, *P < 0.05, significantly different as indicated.

We then used a rheometer to characterize the mechanical properties of the hydrogels. As shown in Figure S10-12, at a concentration of 1.0 wt%, the storage modulus (G') was about 1000 Pa for hydrogels 1-3, respectively, which was significantly higher than that of hydrogel 4 (100 Pa) in Figure S13. Therefore, it can be concluded that the mechanical strength of hydrogel 4 is significantly lower than that of hydrogels 1-3, suggesting that the arrangement of amino acid sequence is crucial to its gelation. Meanwhile, the four hydrogels exhibited weak frequency dependence at the range of 0.1-100 rad s^-1^, indicating that the hydrogels had good elasticity. The storage modulus (G') of the four hydrogels was approximately an order of magnitude higher than their loss modulus (G''), which indicated the formation of true gels. ApoE^-/-^ mice were then subcutaneously (*s.c.*) injected with 100 μL of the different hydrogels or natural IGF-1 protein solution per week and simultaneously fed a high-fat diet for 16 weeks. Atherosclerotic lesion formation was assessed after 16 weeks. Oil Red O staining of *en face* aorta revealed a significant ~42, ~53, and ~27% reduction in lesion area, and there was a corresponding ~53, ~61, and ~33% decrease in plaque size at the aortic root in IGF-1, H1, and H2 treated mice, respectively (Figure 2A-D). Taken together, these data showed that H1 inhibited the development of atherosclerosis.

### The IGF-1 mimetic peptide enhances plaque stability* in vivo*

**Figure 3:**
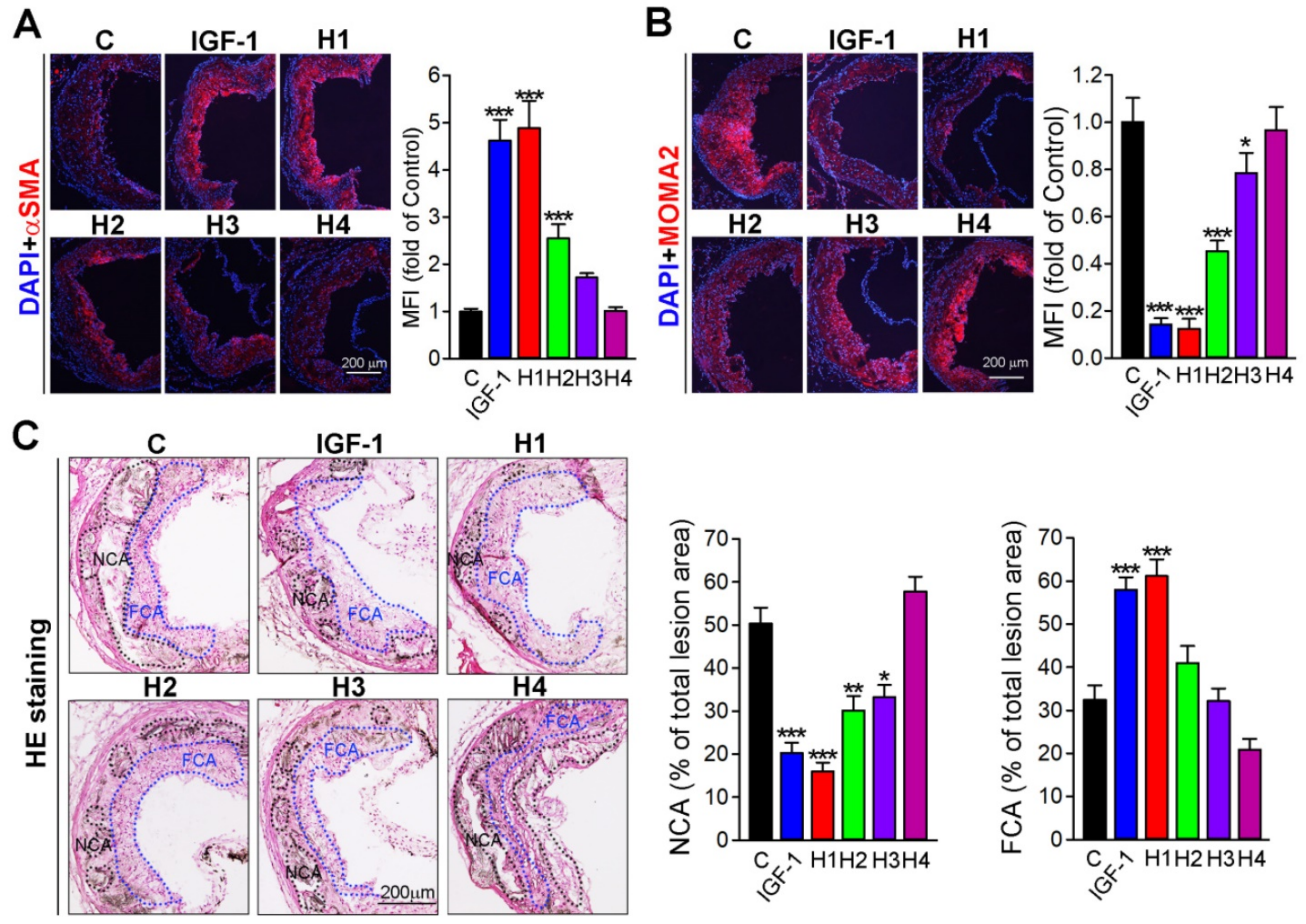
** Analysis of plaque stability.** (**A-B**) Representative photomicrographs of aortic root cross sections from apoE^-/-^ mice followed by immunofluorescent staining for expression of αSMA and MOMA2 with quantification of positive areas, n = 15. (**C**) The aortic root cross sections were assessed by the H&E staining followed by quantitative analysis of necrotic core area and fibrous cap area, n=15. NCA: necrotic cores area marked by a black dashed line; FCA: fibrous cap area marked by a blue dashed line. Data are presented as mean ± SEM, *P < 0.05, **P < 0.01, ***P < 0.001, indicates significant difference from the control.

In atherosclerotic plaque, necrotic core mainly contains lipid and other content, such as calcium, and apoptotic cells residual, which is covered by the fibrous cap that mainly consist of vascular smooth muscle cells (VSMCs) 48. An increase of VSMCs in their number can partially enhance the stability of plaque. Conversely, macrophages can further transform into foam cells, an increase of which can destabilize plaque. Therefore, we analyzed the plaque composition by immunofluorescence staining with αSMA and MOMA2 antibody, markers of VSMC and macrophage, respectively. Our results showed that H1 increased αSMA expression but decreased MAMO2 expression, whose effect was comparable to that of natural IGF-1 protein and better than that of H2, H3, and H4 (Figure 3A-B). Furthermore, we evaluated the stability of plaque by analyzing hematoxylin and eosin (H&E) staining. The results showed that aortic root in mice treated with H1 had higher fibrous cap ratio but lower necrotic core percentages in comparison to that of H2, H3, H4, and IGF-1-treated mice (Figure 3C), indicating that H1 significantly reduced plaque vulnerability. Collectively, H1 enhanced the stability of plaque by increasing the content of fibrous cap and decreasing the necrotic cores; these effects were comparable to natural IGF-1 protein, by which IGF-1 mimetic peptide may reduce the risk of plaque rupture.

### The IGF-1 mimetic peptide reduces lipid accumulation in macrophages and inhibits VSMCs transformation into macrophage-like phenotype

**Figure 4:**
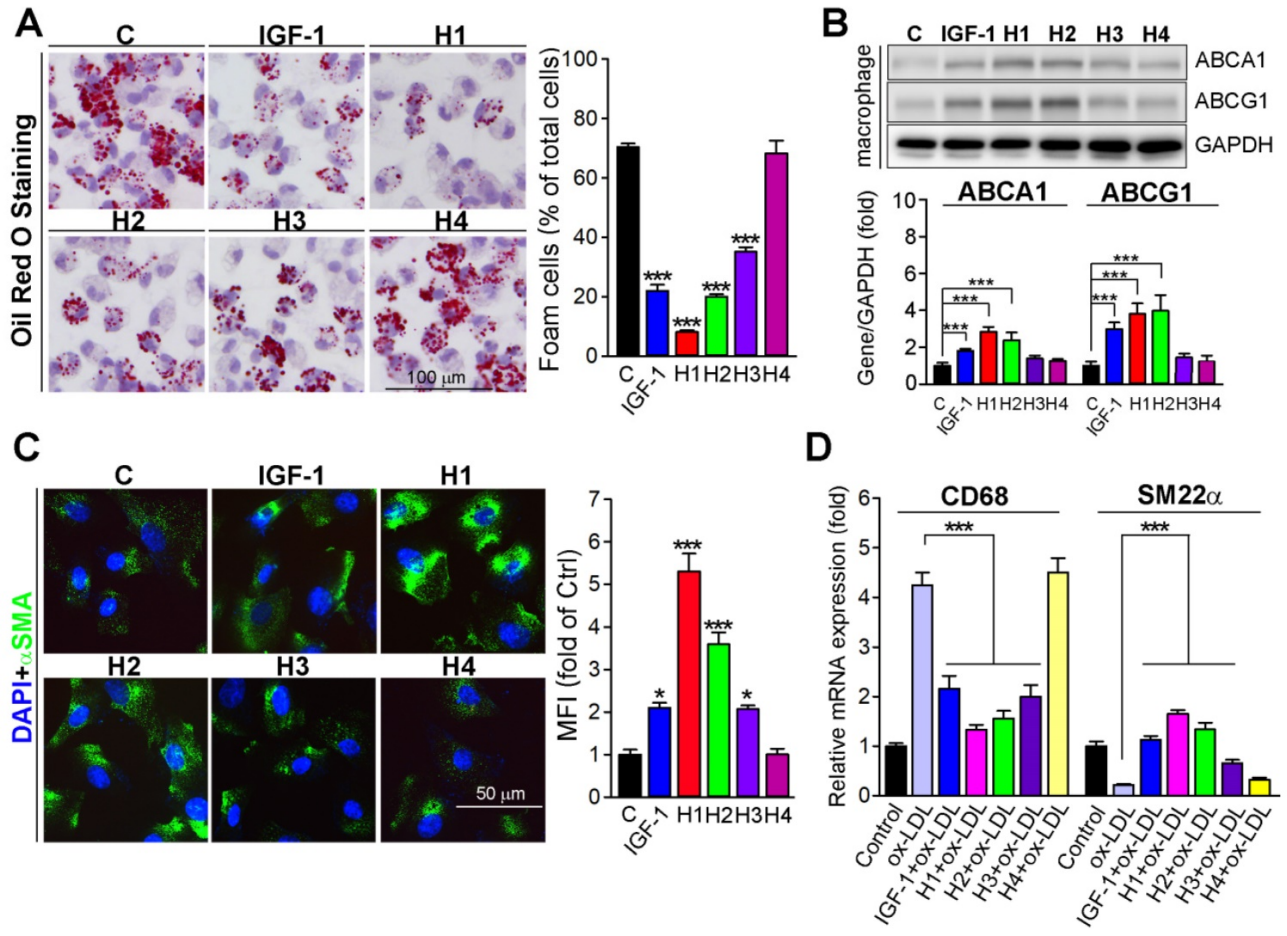
** IGF-1 mimetic peptide inhibits the foam cell formation *in vitro*.** (**A**) Peritoneal macrophages collected from apoE^-/-^ mice were stained with Oil Red O to assess formation of foam cells (>10 lipid droplets per cell, >10 fields per sample), n = 5. (**B**) Expression of ABCA1 and ABCG1 in total cellular extract was determined by western blot after being treated with or without 10 nM of diluted gel with H1, H2, H3, H4 and IGF-1 protein overnight. Quantification of the relative levels of ABCA1 or ABCG1 versus GAPDH in each sample was determined by ImageJ, as a fold of control, n = 5. (**C**) HASMCs were treated with 10 nM of diluted gel with H1, H2, H3, H4 and IGF-1 protein overnight. αSMA expression was analyzed by immunofluorescent staining with quantitation of αSMA-positive cells, n=5. (**D**) HASMCs were treated with 10 nM of diluted gel with H1, H2, H3, H4 and IGF-1 protein in the absence or presence of ox-LDL (50 µg·mL^-1^) overnight. Expression of CD68 and SM22α was determined by qRT-PCR, n = 5. Data are presented as mean ± SEM, *P < 0.05, **P < 0.01, ***P < 0.001 significantly different from control group or as indicated.

Macrophage accumulation of modified LDL leads to foam cell formation, which is a hallmark of atherosclerosis 49. Previous studies indicated that IGF-1 deficiency can result in foam cell formation 50. Therefore, we evaluated the protective effects of H1, H2, H3, H4, and IGF-1 protein against macrophage-derived foam cell formation. We treated peritoneal macrophages with H1, H2, H3, H4 or IGF-1 protein at the same molar concentration. Comparable to the results *in vivo* (Figure 2C and Figure 3B), the *in vitro* results clearly showed that H1 significantly reduced lipid burden in macrophages, which was assessed by Oil Red O staining after exposure to ox-LDL (Figure 4A). Intriguingly, the results obtained after H1 treatment was most effective (8%) compared to that of H2, H3, H4, and natural IGF-1 protein (Figure 4A). The number of foam cells in the group treated with IGF-1 protein, H2, and H3 was reduced to 22.0%, 19.0%, and 37.0%, respectively. There was almost no decrease in intracellular lipid droplets in the group treated with H4 (Figure 4A). Cholesterol efflux from cells to extracellular lipid acceptors plays a vital role in inhibiting lipid accumulation and subsequent foam cell formation 49, 51. Therefore, we assessed the effect of supramolecular nanofiber on cholesterol efflux and found that H1 significantly promotes the cholesterol efflux from peritoneal macrophage (Figure S14). In addition, the effect of Npx and IGF-1 on cholesterol efflux was determined and the promotive effect of IGF-1 on cholesterol efflux was more effective than Npx (Figure S15B), indicating that IGF-1 plays dominant role in promoting cholesterol efflux. To gain insights into potential mechanisms, we assessed expression levels of ABCA1 and ABCG1, which were major lipid-transporters responsible for cholesterol efflux from cells to apolipoproteins 52, 53. Strikingly, we found that administration of IGF-1 protein, H1, and H2 significantly enhanced expression of ABCA1 and ABCG1 in macrophages (Figure 4B), which accounted for the reduction of lipid droplets in ox-LDL treated cells. The phenotypic transformation of VSMC such as transformation into macrophage-like cells, which is also a source of foam cell formation, is the key pathological basis of atherosclerosis. To determine whether IGF-1 mimetic peptide could inhibit the switch of VSMCs into a macrophage-like phenotype, we treated VSMCs with different kinds of IGF-1 mimetic peptides or IGF-1 protein. The immunofluorescent staining results showed that VSMCs treated with IGF-1, H1, H2, and H3 expressed more α-SMA (Figure 4C) than the control expressed. Moreover, VSMCs treated with IGF-1, H1, H2, and H3 expressed more SM22α and less CD68 than the ox-LDL-treated cells did (Figure 4D), suggesting that IGF-1 mimetic peptide could protect VSMCs from transformation into macrophage-like cells. Taken together, IGF-1 mimetic peptide reduces the macrophage and VSMC-derived foam cells, which contributes to the inhibition of atherosclerosis.

### The IGF-1 mimetic peptide imparts anti-inflammatory properties to macrophages *in vitro*

**Figure 5:**
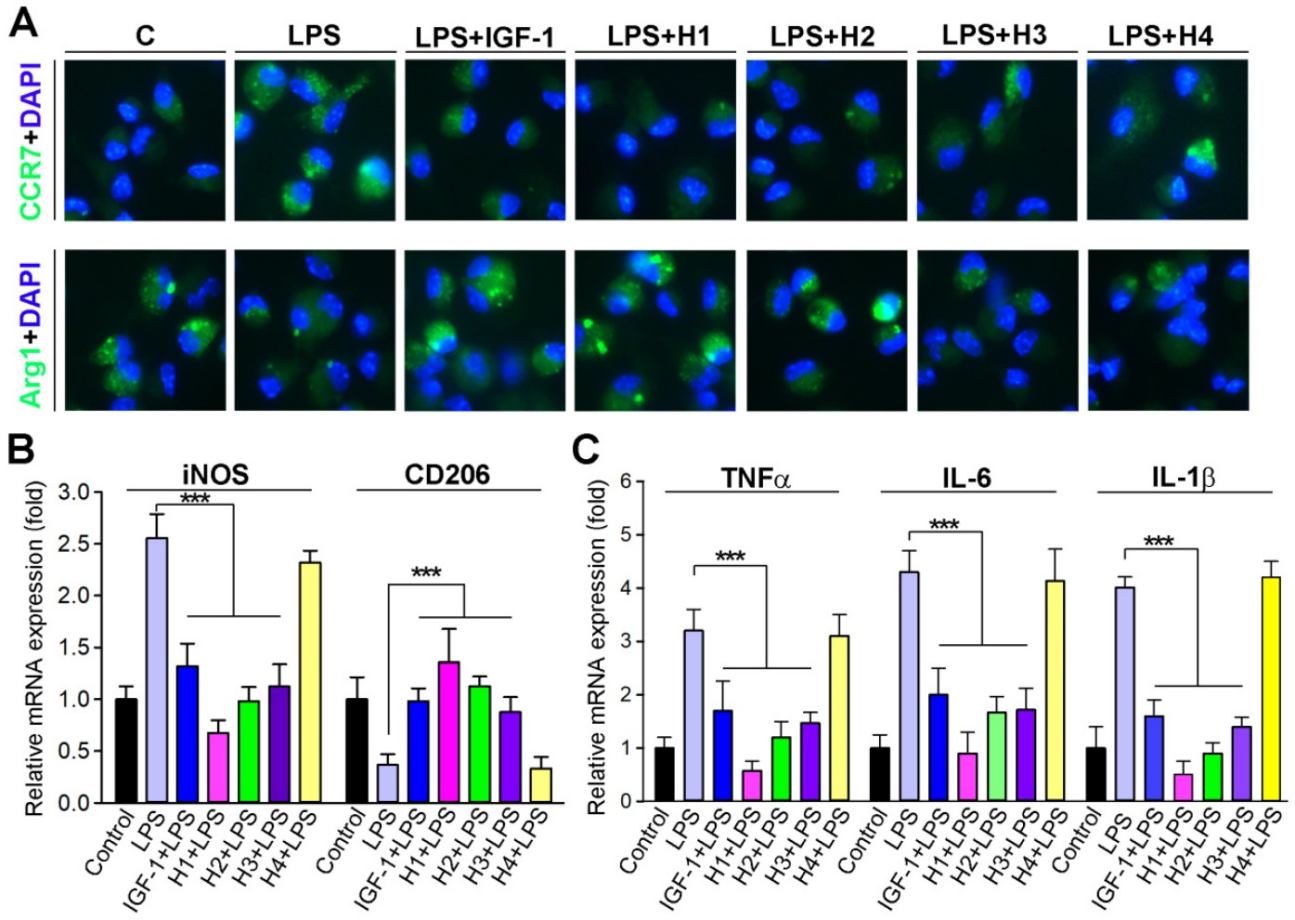
** IGF-1 mimetic peptide inhibits inflammation and promotes the macrophage M2 transition *in vitro*.** (**A**) Peritoneal macrophages collected from apoE^-/-^ mice were pretreated with LPS (100 ng·mL^-1^) for 2 h, followed by addition of 10 nM of diluted gel with H1, H2, H3, H4 or IGF-1 protein overnight. CCR7 and Arg1 expression were determined by immunofluorescent staining with quantitation of CCR7 or Arg1-positive cells, n = 5. (**B-C**) Peritoneal macrophages were treated with 10 nM of diluted gel with H1, H2, H3, H4 or IGF-1 protein in the absence or presence of LPS (100 ng·mL^-1^) overnight. Expression of iNOS and CD206 (B), TNFα, IL-1β and IL-6 mRNA (C) was determined by qRT-PCR, n=5; Data are presented as mean ± SEM, *P < 0.05, **P < 0.01, ***P < 0.001, significantly different as indicated.

The phenotype of macrophages is a critical determinant of atherosclerosis 54. Macrophages play a predominant role in the inflammatory status of atherosclerotic lesions 55; thus, effective intervention in the inflammatory processes of atherogenesis can reduce lesion formation and/or progression. Therefore, it is meaningful to determine whether IGF-1 regulates macrophage function, particularly inflammatory activity. Studies have reported that IGF1R knock out in macrophages results in a reduction in transcripts associated with M2-like macrophage activation 56. Therefore, we examined whether IGF-1 mimetic peptide influenced macrophage polarization. The expression of pro-inflammatory and anti-inflammatory cytokines was assessed in peritoneal macrophages isolated from mice in different groups. We observed that macrophages treated with H1, H2 gels and IGF-1 protein solution expressed less CCR7, whereas more Arg1 compared to control, indicating that H1 could promote the transformation of macrophages to M2 phenotype similarly to IGF-1 (Figure 5A). Moreover, macrophages were treated with lipopolysaccharide (LPS) concurrently with different hydrogels or with IGF-1 protein. The results showed that LPS exposure significantly induced the expression of iNOS, IL-1β, IL-6, and TNFα, markers of inflammation and M1 macrophage phenotype (Figure 5B-C), and inhibited the expression of CD206, a marker of M2 macrophage phenotype. Intriguingly, H1 effectively antagonized the expression of the abovementioned LPS-induced cytokines at the transcriptional level (Figures 5B-C), indicating that H1 exerted an anti-inflammatory effect and imparted anti-inflammatory properties to macrophages. In the supramolecular nanofiber, the anti-inflammatory drug naproxen (Npx) and IGF-1 mimetic peptide co-exists. To demonstrate whose effect counts more, we treated macrophage with Npx, IG1F-1 mimetic peptide, H1, and IGF-1 in presence of LPS. After treatment, we determined the anti-inflammatory effect by evaluating transcriptional levels of anti-inflammatory cytokines. The results showed that both naproxen and IGF-1 mimetic peptide partly blocked LPS-induced inflammation. Noticeable, the anti-inflammatory effect of naproxen was more effective than that of IGF-1 mimetic peptide; but is less effective than that of H1 (Figure S15A). These results indicated that naproxen plays dominant role in anti-inflammatory function while IGF-1 mimetic peptide plays minor role; and IGF-1 mimetic peptide may enhance the anti-inflammatory effect of naproxen, which account for the more anti-inflammatory effect of H1. Collectively, our results demonstrated that H1 efficiently promoted the macrophage transition to displaying an anti-inflammatory phenotype and inhibiting the expression of proinflammatory cytokines *in vitro*, which partially account for the benefit to atherosclerosis treatment.

### The IGF-1 mimetic peptide inhibits inflammation and imparts anti-inflammatory properties to macrophages* in vivo*

**Figure 6:**
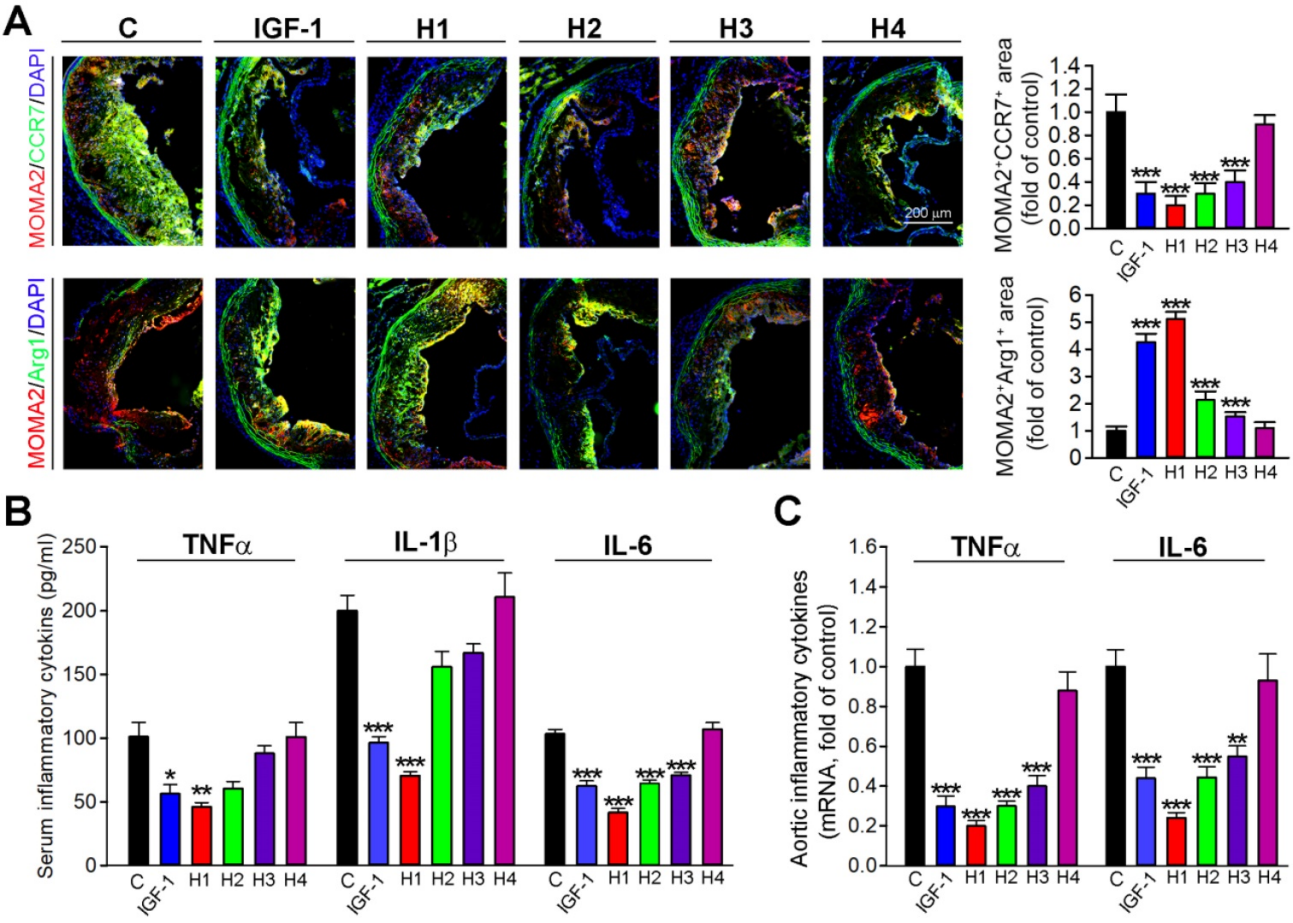
** IGF-1 mimetic peptide inhibits inflammation and promotes the macrophage M2 transition *in vivo*.** (**A**) Aortic root cross sections visualized by co-immunofluorescent staining with anti-CCR7 or Arg1 and MOMA2 antibodies with quantitative analysis of MOMA2^+^ CCR7^+^ or MOMA2^+^ Arg1^+^ areas, n=15. (**B**) Serum TNF-α, IL-β and IL-6 were determined by ELISA, n=8. (**C**) qRT-PCR analysis of TNF-α and IL-6 in aortas, n=8. Data are presented as mean ± SEM, *P < 0.05, **P < 0.01, ***P < 0.001, significantly different from control group.

To further determine whether H1, H2, H3, H4 and IGF-1 protein could inhibit the inflammatory response *in situ*, we evaluated the expression of macrophage phenotypic markers in frozen sections of aortic root by double immunofluorescence staining. Consistent with *in vitro* observations, the results obtained *in vivo* demonstrated that H1 and IGF-1 administration induced the expression of Arg1, an M2 macrophage marker; while inhibited the expression of CCR7, an M1 macrophage marker (Figure 6A); these results indicated that H1 could polarize macrophages and cause them to display an anti-inflammatory phenotype similarly to IGF-1. To further determine mechanisms of the antiatherogenic effect of natural IGF-1 and IGF-1 mimetic peptides, we measured circulating levels of IL-6, IL-1β and TNFα, the proinflammatory cytokines involved in atherosclerotic lesion development. *In vivo*, these proinflammatory cytokines in the plasma were reduced in IGF-1 and H1-treated apoE^-/-^ mice compared with the control group (Figure 6B). Furthermore, we detected the expression of proinflammatory cytokines in aortas. The data showed that H1, H2, H3, and IGF-1 protein reduced the expression of these cytokines, among which the H1 had the highest anti-inflammatory activity (Figure 6C). Taken together, our data demonstrated that H1 could promote the macrophage M2 transition, impart anti-inflammatory properties, and downregulate expression of vascular proinflammatory cytokine genes *in vivo*, thereby, partially inhibiting the progression of atherosclerosis.

## Discussion

Atherosclerosis, a disease of lipid disorder and chronic inflammation, is the major pathogeny of cardiovascular disease that caused leading morbidity and mortality worldwide. Researches have shown that lower circulating IGF-1 levels are associated with an increased risk of atherosclerosis 7, 57. Moreover, IGF-1 or IGF-1 receptor deficiency can accelerate atherosclerosis progression 50, 58; yet, exogenous IGF-1 infusion in Apoe^-/-^ mice can reduce atherosclerotic plaque size and accumulation of foam cells in plaque 59. Thus, increasing IGF-1 level in circulation might serve as a promising therapeutic approach for atherosclerosis treatment. Based on the pathogenesis of atherosclerosis, herein, we designed supramolecular nanofibers that qualified with anti-inflammatory property and IGF-1 bioactivity. Furthermore, we demonstrated that IGF-1 mimetic peptides could diminish systematic inflammation and promote the cholesterol efflux of macrophages through induction of ABCA1 and ABCG1, by which inhibiting foam cell formation and the development of atherosclerotic lesions.

Foam cell formation from macrophages and VSMC is a hallmark of atherosclerosis 3. Previous studies reported that IGF1R deficiency in macrophages reduced expression of cholesterol transporters, thereby decreased cholesterol efflux, which in turn resulted in foam cell accumulation in lesions, increased atherosclerotic burden 50. In our study, IGF-1 or IGF-1 mimetic peptides markedly reduced macrophage-derived foam cell accumulation in plaque (Figure 1F). In addition, macrophages that treated with IGF-1 or IGF-1 mimetic peptides showed less lipid burden than control (Figure 3A), indicating that supplementation of IGF-1 could inhibit the foam cell formation. To determine mechanisms whereby IGF-1 reduces lipid accumulation in macrophages, we assessed the expression of cholesterol transporters, such as ABCA1 and ABCG1, which facilitated the cholesterol efflux. Noticeable, IGF-1 or IGF-1 mimetic peptides significantly promoted the expression of both ABCA1 and ABCG1 (Figure 3B), which accounted for the inhibition of foam cell formation.

Clinically, most acute coronary events result from erosion or rupture of atherosclerotic plaques 60. Unstable plaques that are prone to rupture have characteristic of a thin fibrous cap with decreased number of vascular smooth muscle cells (VSMCs) 61, 62. Therefore, plaque stabilization is a critical determinant of cardiovascular events. VSMCs is prominent content of atherosclerotic plaque, the phenotype and content of which determines the stability of plaque 63. It is reported that overexpression of IGF-1 in VSMCs or IGF-1 treatment increased features of plaque stability by altering VSMC phenotype 64, 65. In our study, IGF-1 mimetic peptides increased the VSMCs content in plaque (Figure 3A), indicating that plaque stability was enhanced. *In vitro*, IGF-1 mimetic peptides markedly increased the expression of SM22α, the marker of SMC; whereas decreased the CD68, the marker of macrophage (Figure 4C-D), which suggested that IGF-1 mimetic peptides could prevent the VSMCs against switch to macrophage-like SMCs, the contributor to plaque formation.

Inflammatory response and associated macrophage polarization (M1) are important contributors to development of atherosclerosis. Previous study has reported that IGF-1 can reduce inflammatory responses, suppress oxidative stress, and decrease atherosclerosis progression in ApoE-deficient mice 13. Therefore, we determined whether IGF-1 mimetic peptides could inhibit the inflammatory response during the development of atherosclerosis. The data showed that H1 significantly promoted the macrophage transformation to M2 phenotype *in vitro* (Figure 5A-B). Moreover, *in vitro* experiment, H1 also reduced the production of proinflammatory cytokines in macrophages at transcriptional level (Figure 5A-B). Furthermore, we observed that H1 favored the switch of macrophages in plaque to M2 polarization (Figure 6A), indicating that inflammatory response was reduced by H1. Indeed, the anti-inflammatory effect of H1 was further confirmed by quantitation of proinflammatory cytokines in serum and aortas at transcriptional level (Figure 6C-D). The excellent anti-inflammatory effect of H1 may be attributed to its capping group naproxen (Npx), a clinical drug for inhibiting inflammation 47.

Dysfunction of lipid metabolism is a significant contributor to atherosclerosis development. However, in this study, lipid profile and body weight of the mice were not changed by IGF-1 mimetic peptides (Table 2), indicating that the anti-atherogenic effect of IGF-1 mimetic peptides is independent of regulating lipid metabolism and other properties, such as anti-inflammation, promoting cholesterol efflux, and inhibiting formation of macrophage-like SMCs, act as major contributors to its anti-atherogenic effect. Taken together, we designed a bifunctional supramolecular nanofiber that combined the bioactivity of IGF-1 and the anti-inflammatory property of Npx to effectively inhibit the development of atherosclerosis. Our study provides a promising nanomedicine for the treatment of atherosclerosis. Besides, the integration of multi-functions in supramolecular nanomaterials of peptides may lead to the outcome of multifunctional nanomedicines for the treatment of various diseases in the future.

## Materials and Methods

### Synthesis of peptide

All peptide derivatives were synthesized by the standard solid phase peptide synthesis (SPPS). We purchased 2-chlorotriacyl chloride resin and amino acids whose N-terminal is protected by Fmoc and side chain is properly protected. Firstly the C-terminal of the first amino acid was conjugated on the resin. Anhydrous N,N'-dimethyl formamide (DMF) containing 20% piperidine was used to remove Fmoc protected group. To couple the next amino acid to the free amino group, O-Benzotriazol-1-yl-N,N,N',N'-tetramethyluronium hexafluorophosphate (HBTU) was used as coupling reagent. Peptides chain was extended according the standard SPPS protocol. Naproxen (Npx-) was used at the final step as capping group. Lastly, 95% TFA containing 2.5% H_2_O and 2.5% TIS was used to cleave peptides derivative from resin and the mixture was filtered. Ice cold diethylether was poured into filtrate concentrated by rotary evaporation. The precipitate was centrifuged for 5 min at a speed of 5000 rpm. The solid was dried by vacuum pump and then purified by high-performance liquid chromatography (HPLC) to obtain the pure compounds.

### Preparation of hydrogels

Assembly into hydrogels was achieved utilizing a simplistic heating-cooling process. In brief, 2.5 mg of compound was dissolved in 496 μL of PBS along with 4 μL of 1 M sodium carbonate, and the pH was adjusted to 7.4. The suspension was heated with an alcohol lamp until completely dissolved. Hydrogel formation was observed after the suspension was heated and cooled for 5 minutes.

### Microscale thermophoresis

rhIGF-1R was labeled with the fluorescent dye NT-647 using a Monolith NT™ Protein Labelling Kit (cysteine-reactive) (NanoTemper Technologies, Germany). PBS buffer containing 0.05% Tween 20 (pH 7.4) was used as the assay buffer. For the interaction experiments of fluorescent proteins with Compound ***1, 2, 3***or ***4***, the concentration of labeled proteins were kept constant, while the concentration of Compound ***1, 2, 3***or ***4*** varied from 0.054 nM to 2 μM. Then, the solution of fluorescent proteins was mixed with solutions containing different concentrations of Compound ***1, 2, 3***or ***4*** at 1:1 volume ratio. After a short incubation time, the samples were loaded into MST NT.115 standard glass capillaries and the analysis was performed using the Monolith NT.115 system (NanoTemper Technologies, Germany). The K_D_ value was calculated using the NanoTemper software package.

### Cell culture

Human aortic smooth muscle cells (HASMCs) were cultured in a completed DMEM F12 medium (10% fetal bovine serum, 50 μg/mL penicillin, and 50 μg/mL streptomycin). Peritoneal macrophages were cultured in complete RPMI1640 medium (10% fetal bovine serum, 50 μg/mL penicillin, 50 μg/mL streptomycin and 2 mM glutamine). HASMCs or peritoneal macrophages were seeded on a 6-well plate at the density of 3×10^5^ cells per well before treatment. Then, the cells were cultured for 16 hours with IGF-1 protein (10 nM), diluted H1 (10 nM), H2 (10 nM), H3 (10 nM), or H4 (10 nM).

### Determination of Protein or mRNA Expression

After treatment, total cellular proteins were extracted from peritoneal macrophages for determination of ABCA1 and ABCG1 by western blot, as previously described 66. Expression of CD68, SM22α, iNOS, CD206, TNF-α, IL-6, and IL-1β mRNA in HASMCs or macrophages was determined by quantitative real-time polymerase chain reaction (qRT-PCR) with total RNA extracted from cells and the primers listed in Table 1 and normalized to GAPDH mRNA levels in the corresponding samples.

### Determination of foam cell formation

To assess foam cell formation *in vitro*, peritoneal macrophages collected from apoE^-/-^ mice were plated on cover slips in 12-well plates. Subsequently, induction and determination of foam cells was conducted as previously described 66.

### *In vivo* studies

All animal studies were approved by the Animal Ethics Committee of Nankai University and followed the Tianjin Committee of Use and Care of Laboratory Animals. Male apoE^‑/‑^ mice (~22 g bodyweight, ~8 weeks old) were purchased from the Beijing Vital River Laboratory Animal Technology Co., Ltd, which were used to set up the atherosclerotic model, as previously described^66^. ApoE^-/-^ mice were randomly assigned into six groups (15 per group) and fed with HFD (21% fat plus 0.5% cholesterol; Mediscience, Ltd, Jiangsu, China; Cat No. MD12015A) for 16 weeks. ApoE^-/-^ mice in the different groups were subcutaneously injected with either saline (100 µL, n=15), IGF-1 protein (1 μM, 100 μL, n=15), H1 (1 μM, 100 μL, n=15), H2 (1 μM, 100 μL, n=15), H3 (1 μM, 100 μL, n=15) or H4 (1 μM, 100 μL, n=15) weekly. At the end of the experiment, all mice were euthanatized by an overdose of 2,2,2-tribromoethanol (640 mg/kg, IP injection), followed by collection of aortas, blood, and peritoneal macrophages. Serum was prepared to determine levels of TNF-α, IL-1β and IL-6 by ELISA kits purchased from ABclonal, Inc. (Wuhan, China). The *en face* aortas were used to prepare aortic root cross sections followed by determination of *en face* and sinus lesions with Oil Red O staining. All the images were obtained with a microscope and quantified lesion areas in en face aorta by a computer-assisted image analysis protocol (Photoshop CS6). Necrotic core area, fibrous cap area, expression of MOMA-2, CD68, and α-SMA protein in lesion were determined by H&E and immunofluorescent staining with aortic root cross sections.

### Reagents

Rabbit anti-ABCG1, ABCA1 polyclonal antibodies were purchased from Novus Biologicals (Littleton, CO). Mouse anti-Arg1, α-SMA, SM22α, and CD68 monoclonal antibody were purchased from Santa Cruz Biotechnology, Inc. (Santa Cruz, CA). Rabbit anti-CCR7 polyclonal antibodies were purchased from ABclonal. Rabbit anti-iNOS polyclonal antibodies were purchased from Proteintech Group, Inc. (Rosemont, IL). Mouse anti-rabbit IgG-R, mouse anti-rabbit IgG-FITC and m-IgGκ BP-FITC antibodies were purchased from Santa Cruz Biotechnology, Inc. (Santa Cruz, CA).

### Statistical analysis

The data and statistical analysis comply with the recommendations on experimental design and analysis in pharmacology 67. All data are expressed as mean ± SEM or mean ± SD. One-way ANOVA for comparisons between multiple groups followed by Turkey's method. Significance was accepted when *P* < 0.05.

## Supplementary Material

Supplementary figures and tables.

## Figures and Tables

**Table 2:** Body weight (BW) and serum lipid profile in apoE^-/-^ mice^†^

	Control	IGF-1	H1	H2	H3	H4
*BW(g)*	26.25±1.3	25.04±1.83	26.49±2.14	25.55±2.31	26.79±1.77	26.81±2.06
*Total-C*	16.31±1.84	15.47±2.76	15.06±2.08	14.76±2.61	14.74±2.55	16.63±2.05
*HDL-C*	2.48±0.76	2.35±0.89	2.41±0.25	2.41±0.43	2.4±0.28	2.34±0.76
*LDL-C*	4.03±1.32	4.08±1.42	4.35±0.77	4.53±1.58	4.27±0.39	4.45±1.23
*TG*	0.49±0.14	0.51±0.13	0.5±0.2	0.49±0.15	0.5±0.2	0.52±0.08

†: Male apoE^-/-^ mice were treated as indicated in Figure 2. Serum samples were prepared to determine the levels of total cholesterol (Total-C), LDL- and HDL-C, and triglyceride (TG, mM). Date are presented as mean ± SD (n=15), *P<0.05 *vs*. control.

**Table 1:** Sequences of primers for q-RT-PCR

GENE	Forward	Backward
*Mus IL-1β*	GAAATGCCACCTTTTGACAGTG	TGGATGCTCTCATCAGGACAG
*Mus IL-6*	CTGCAAGAGACTTCCATCCAG	AGTGGTATAGACAGGTCTGTTGG
*Mus TNFα*	CAGGCGGTGCCTATGTCTC	CGATCACCCCGAAGTTCAGTAG
*Mus iNOS*	ACATCGACCCGTCCACAGTAT	CAGAGGGGTAGGCTTGTCTC
*Mus CD206*	CTCTGTTCAGCTATTGGACGC	TGGCACTCCCAAACATAATTTGA
*Homo SM22α*	AGTGCAGTCCAAAATCGAGAAG	CTTGCTCAGAATCACGCCAT
*Homo CD68*	GGAAATGCCACGGTTCATCCA	TGGGGTTCAGTACAGAGATGC
*Mus GAPDH*	AGGTCGGTGTGAACGGATTTG	GGGGTCGTTGATGGCAACA
*Homo GAPDH*	GGAGCGAGATCCCTCCAAAAT	GGCTGTTGTCATACTTCTCATGG

## Abbreviations

LPS: lipopolysaccharide
CCR7: Cxc Chemokine Receptor 7
oxLDL: oxidized LDL
α-SMA: α-smooth muscle actin
HASMC: human aortic smooth muscle cells
IL-1β: interleukin-1β
TNF-α: tumor necrosis factor-α
IL-6: interleukin-6
iNOS: inducible nitric oxide synthase
GAPDH: glyceraldehyde-3-phosphate dehydrogenase
CD206: mannose receptor C-type 1
SM22α: smooth muscle 22 α
