# Supplementary material for: Bifunctional supramolecular nanofiber inhibits atherosclerosis by enhancing plaque stability and anti-inflammation in apoE-/- mice
Source: Theranostics. 2020 Aug 13;10(22):10231–44. doi: 10.7150/thno.48410 (PMC7481406; doi:10.7150/thno.48410)
Supplement: Supplementary file 1 — Supplementary figures and tables. [file thnov10p10231s1.pdf]

Supporting Information

Bifunctional supramolecular nanofiber inhibits atherosclerosis by enhancing plaque stability and anti-inflammation in apoE$^{-/-}$ mice

Yuna Shang[1#], Chuanrui Ma[2.3#], Jing Zhang[2], Zhongyan Wang[1.4], Chunhua Ren[1], Xin Luo[1], Rong Peng[1], Jingfei Liu[1], Jingyuan Mao[2], Yang Shi[1]*& Guanwei Fan[2,3]*

[1]State Key Laboratory of Medicinal Chemical Biology, College of Life Sciences, Key Laboratory of Bioactive Materials, Ministry of Education, Collaborative Innovation Center of Chemical Science and Engineering, and National Institute of Functional Materials, Nankai University, Tianjin 300071, P. R. China

[2]First Teaching Hospital of Tianjin University of Traditional Chinese Medicine, Tianjin Key Laboratory of Translational Research of TCM Prescription and Syndrome, Tianjin 300381, P. R. China

[3]State Key Laboratory of Modern Chinese Medicine, Tianjin University of Traditional Chinese Medicine, Tianjin 300193, P. R. China

[4]Tianjin Key Laboratory of Radiation Medicine and Molecular Nuclear Medicine, Institute of Radiation Medicine, Chinese Academy of Medical Sciences & Peking Union Medical College, Tianjin 300192, P.R. China.

Characteristic of compounds:

Npx-^{D}F^{D}FGSSSR: ^{1}H NMR (400 MHz, DMSO-d_6) δ 8.29 (t, *J* = 5.5 Hz, 1H), 8.22 (d, *J* = 8.0 Hz, 1H), 8.12 (m, 5H), 8.01 (d, *J* = 7.8 Hz, 1H), 7.74 (d, *J* = 8.9 Hz, 1H), 7.66 (d, *J* = 8.5 Hz, 1H), 7.59 (s, 2H), 7.29 (s, 5H), 7.24 – 7.20 (m, 3H), 7.16 (dd, *J* = 8.9, 2.3 Hz, 2H), 6.97 – 6.92 (m, 5H), 4.59 (m, 1H), 4.46 (d, *J* = 16.9 Hz, 3H), 4.37 (m, 3H), 4.23 – 4.18 (m, 2H), 3.88 (d, *J* = 5.7 Hz, 4H), 3.83 (d, *J* = 5.2 Hz, 2H), 3.79 (d, *J* = 6.9 Hz, 1H), 3.67 – 3.61 (m, 5H), 3.11 (t, *J* = 8.7 Hz, 3H), 2.88 (m 2H), 2.70 (dd, *J* = 13.5, 9.8 Hz, 1H), 1.82 – 1.72 (m, 1H), 1.65 – 1.58 (m, 1H), 1.53 (m, 2H), 1.35 (d, *J* = 6.9 Hz, 3H). HR-MS: calc. M = 998.4498, obsvd. $(M+H)^+$ = 999.4559.

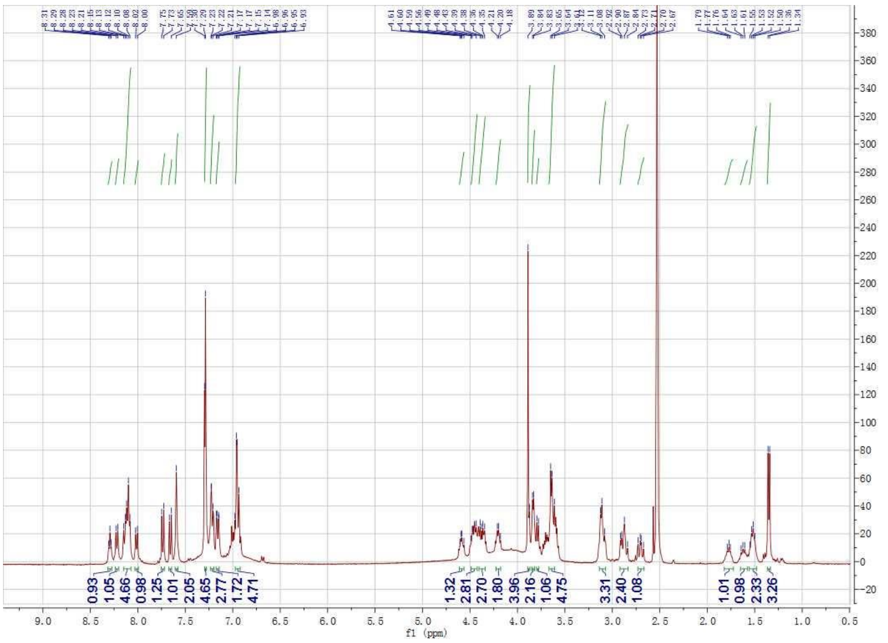

Figure S1. ^{1}H-NMR spectrum of Npx-^{D}F^{D}FGSSSR (compound ***1***).

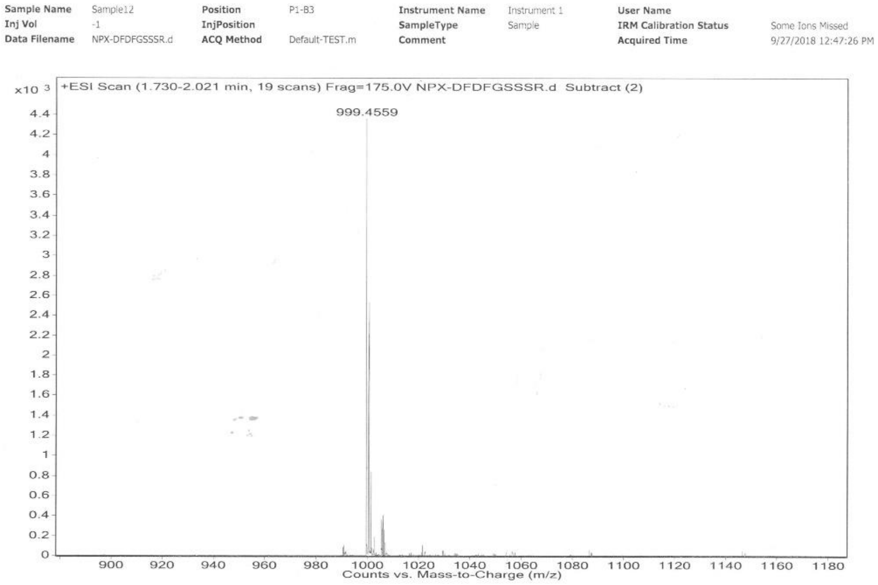

Figure S2. HR-MS spectrum of Npx-^{D}F^{D}FGSSSR (compound ***1***).

Npx-FFGSSSR: ^{1}H NMR (400 MHz, DMSO-d$_6$) δ 8.28 – 8.18 (m, 2H), 8.15 (d, *J* = 7.8 Hz, 2H), 8.09 (s, 2H), 8.04 (d, *J* = 7.5 Hz, 1H), 7.74 (dd, *J* = 15.0, 8.8 Hz, 2H), 7.66 (d, *J* = 6.9 Hz, 2H), 7.36 (d, *J* = 8.2 Hz, 1H), 7.29 (s, 1H), 7.24 (s, 5H), 7.16 (d, *J* = 4.4 Hz, 3H), 7.10 (d, *J* = 6.9 Hz, 2H), 7.04 (d, *J* = 6.8 Hz, 1H), 4.57 (d, *J* = 0.5 Hz, 1H), 4.50 (d, *J* = 3.1 Hz, 1H), 4.41 (s, 2H), 4.37 (s, 2H), 4.23 – 4.17 (m, 1H), 3.87 (s, 4H), 3.80 (s, 3H), 3.75 (d, *J* = 7.2 Hz, 2H), 3.66 – 3.59 (m, 6H), 3.12 (d, *J* = 4.9 Hz, 2H),

3.01 (m, 2H), 2.77 (m, 2H), 1.78 (d, *J* = 6.7 Hz, 1H), 1.64 (m, 1H), 1.52 (d, *J* = 4.7 Hz, 2H), 1.35 (d, *J* = 6.6 Hz, 1H), 1.21 (d, *J* = 6.5 Hz, 3H). HR-MS: calc. M = 998.4498, obsvd. $(M+H)^+$ = 999.4559.

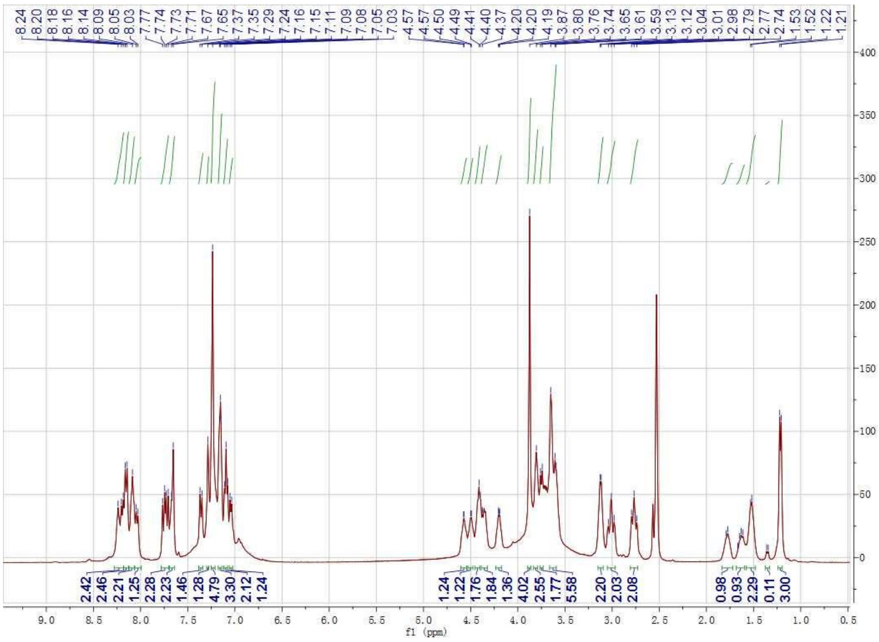

Figure S3. ^{1}H-NMR spectrum of Npx-FFGSSSR (compound ***2***).

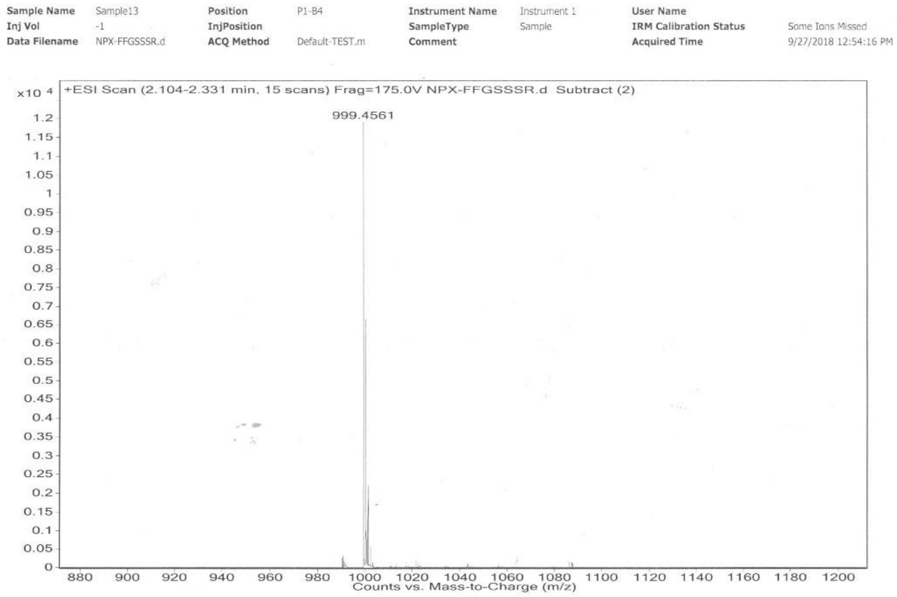

Figure S4. HR-MS spectrum of Npx-FFGSSSR (compound ***2***).

Nap-FFGSSSR: ^{1}H NMR (400 MHz, DMSO-d$_6$) δ 8.26 (s, 1H), 8.13 (m, 4H), 7.96 (d, *J* = 6.3 Hz, 1H), 7.71 (d, *J* = 7.8 Hz, 1H), 7.63 (d, *J* = 7.1 Hz, 1H), 7.54 (d, *J* = 20.1 Hz, 2H), 7.18 (m, 10H), 6.93 (s, 5H), 4.39 (m, 6H), 3.93 – 3.73 (m, 7H), 3.61 (s, 18H), 3.08 (s, 3H), 2.85 (s, 2H), 2.68 (s, 1H), 1.84 – 1.41 (m, 3H), 1.33 (s, 2H). HR-MS: calc. M = 954.4236, obsvd. (M+H)$^+$ = 955.4293.

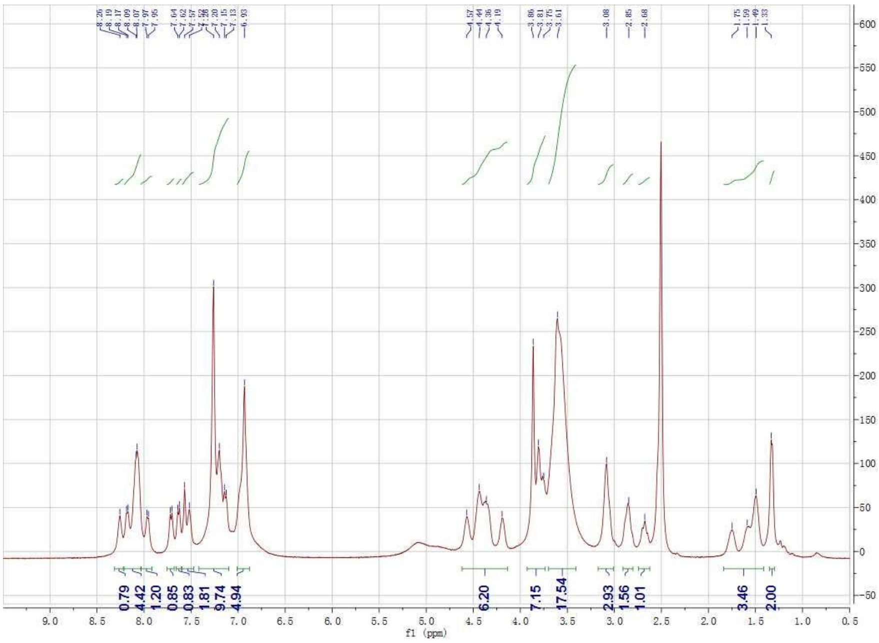

Figure S5. ^{1}H-NMR spectrum of Nap-FFGSSSR (compound ***3***).

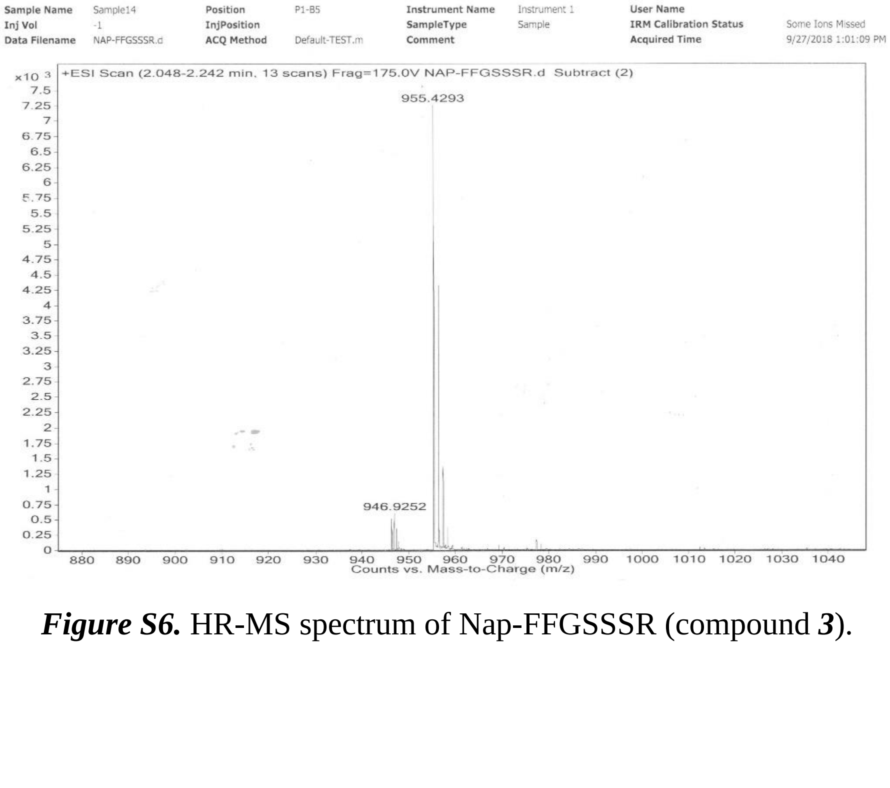

Figure S6. HR-MS spectrum of Nap-FFGSSSR (compound ***3***).

Nap-FFGSRSS: ^{1}H NMR (400 MHz, DMSO-d_6) δ 8.35 (d, *J* = 8.3 Hz, 1H), 8.29 (d, *J* = 6.4 Hz, 2H), 8.22 (d, *J* = 7.9 Hz, 1H), 8.10 (d, *J* = 7.2 Hz, 1H), 8.03 (d, *J* = 7.5 Hz, 2H), 7.88 (d, *J* = 7.6 Hz, 1H), 7.81 (s, 1H), 7.79 (s, 1H), 7.78 (s, 1H), 7.76 (s, 1H), 7.59 (m, 2H), 7.53 – 7.45 (m, 2H), 7.26 (t, *J* = 7.8 Hz, 4H), 7.22 (s, 1H), 7.18 (d, *J* = 7.4 Hz, 7H), 4.63 – 4.51 (m, 3H), 4.40 (m, 4H), 4.31 – 4.26 (m, 2H), 3.83 (s, 2H), 3.75 (m, 1H), 3.66 (d, *J* = 3.6 Hz, 2H), 3.61 (m, 4H), 3.53 (s, 1H), 3.50 (s, 1H), 3.11 (d, *J* = 6.1 Hz, 2H), 3.07 (d, *J* = 4.1 Hz, 1H), 3.01 (d, *J* = 4.0 Hz, 1H), 2.98 (d, *J* = 3.8 Hz, 1H), 2.86 (m, 1H), 2.75 (m, 1H), 1.81 (d, *J* = 7.8 Hz, 1H), 1.61 (d, *J* = 7.0 Hz, 1H), 1.55 (d, *J* = 7.2 Hz, 2H). HR-MS: calc. M = 954.4236, obsvd. $(M+H)^+$ = 955.43.

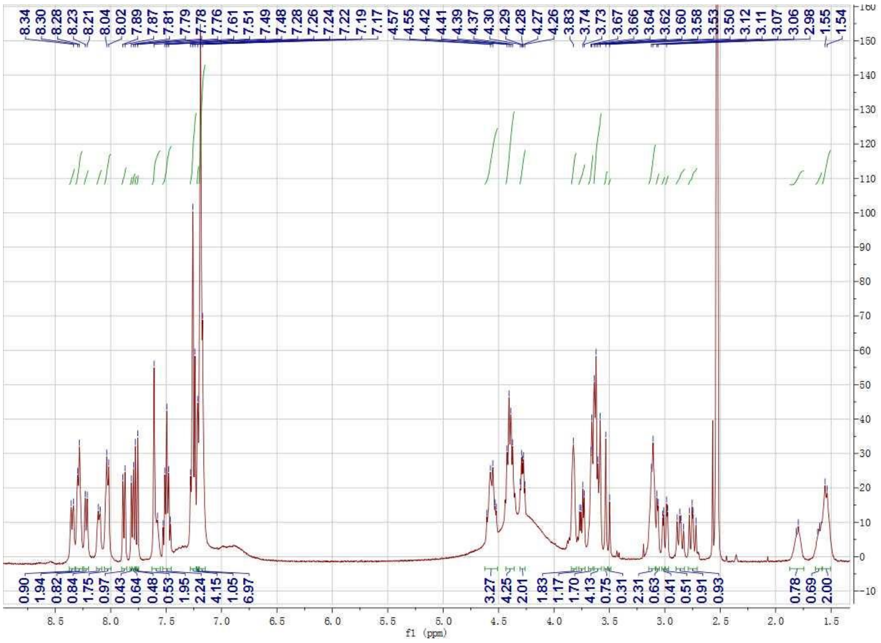

Figure S7. ^{1}H-NMR spectrum of Nap-FFGSRSS (compound ***4***).

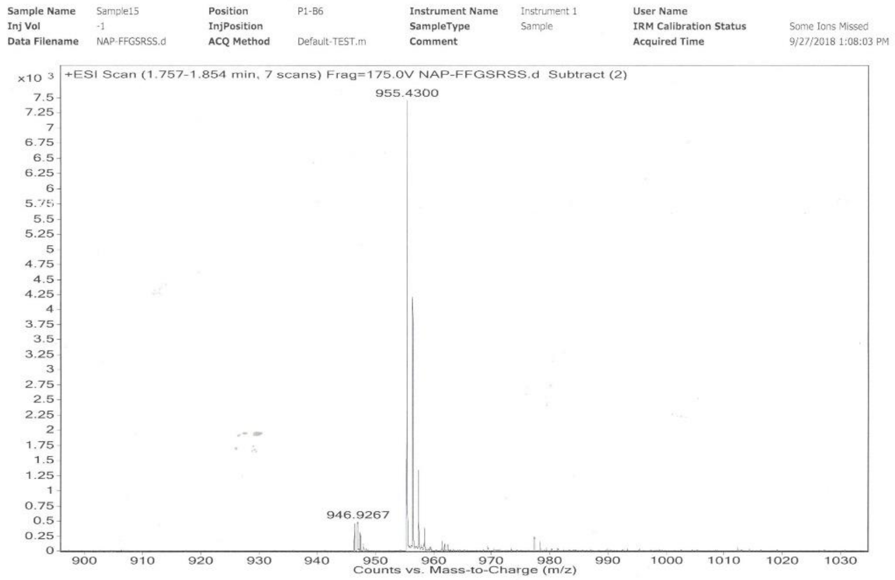

Figure S8. HR-MS spectrum of Nap-FFGSRSS (compound ***4***).

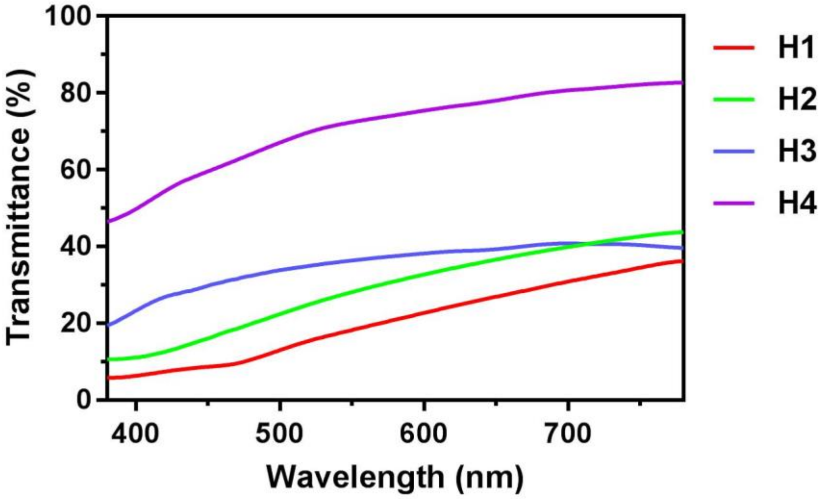

Figure S9. Transmittance of the four hydrogels as a function of the wavelength.

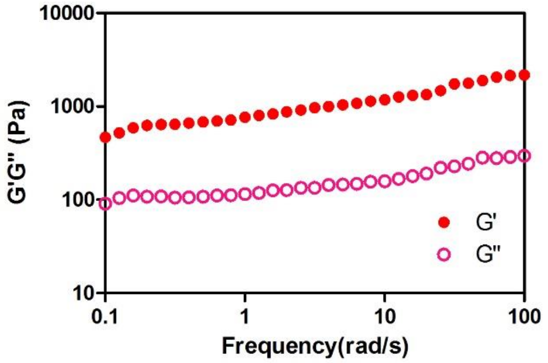

Figure S10. Dynamic frequency sweep of hydrogel 1 (H1) at strain of 1% at 37 ^{o}C

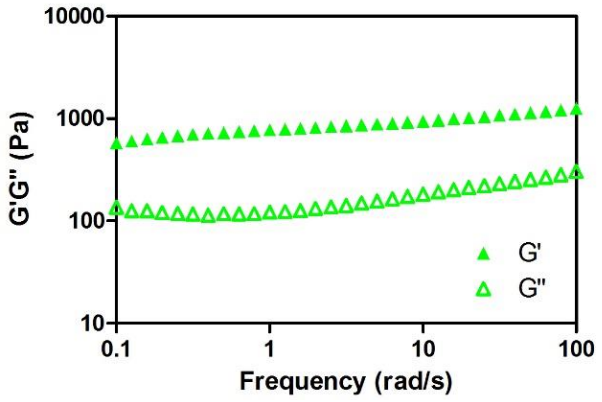

Figure S11. Dynamic frequency sweep of hydrogel 2 (H2) at strain of 1% at 37 ^{o}C

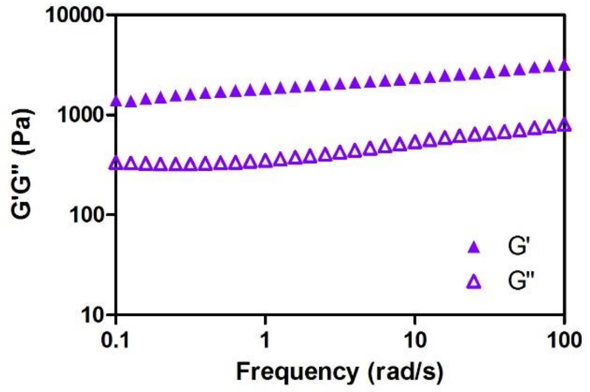

Figure S12. Dynamic frequency sweep of hydrogel 3 (H3) at strain of 1% at 37 ^{o}C

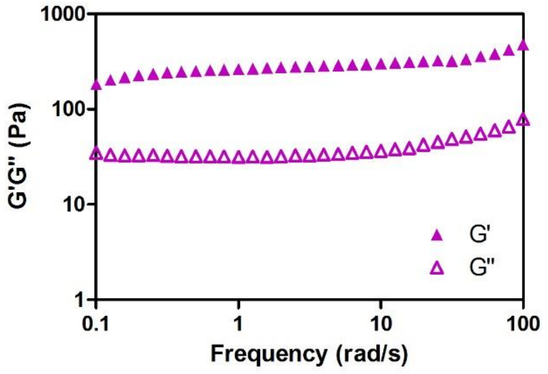

Figure S13. Dynamic frequency sweep of hydrogel 4 (H4) at strain of 1% at 37 ^{o}C

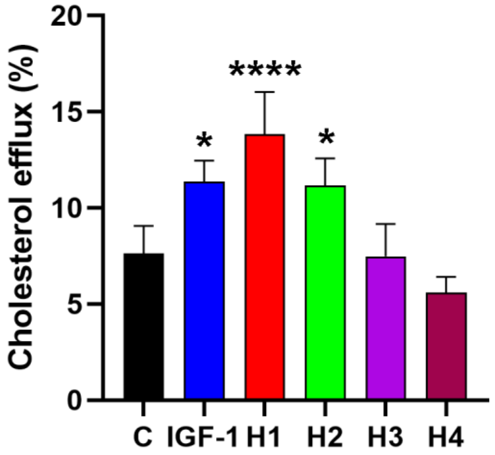

Figure S14. Cholesterol efflux of peritoneal macrophage were assessed after treatment as indicated, n=5. Data are presented as mean ± SEM, *P < 0.05, **P < 0.01, ***P < 0.001, significantly different as indicated.

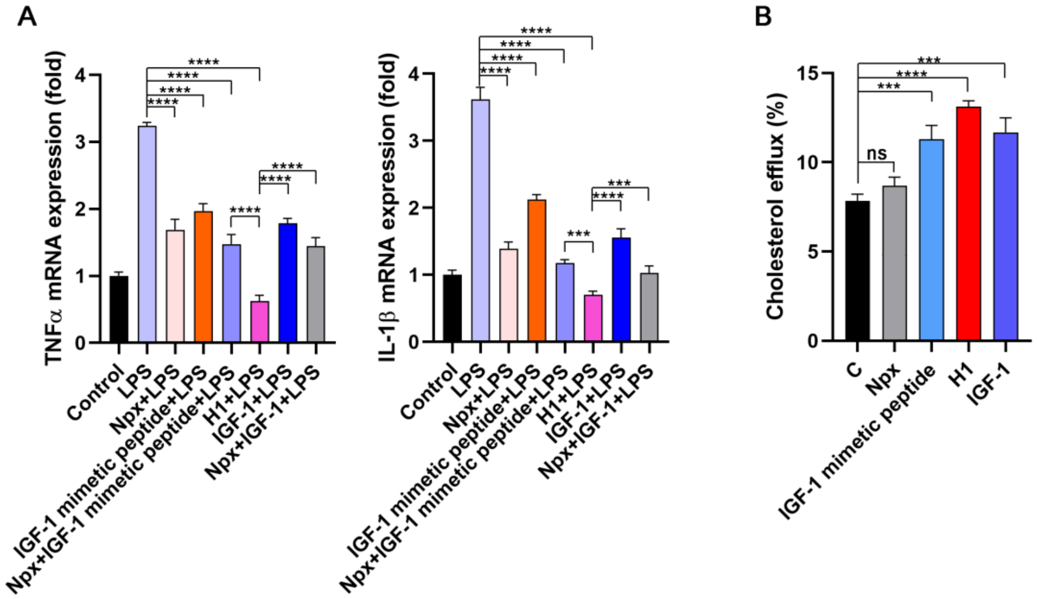

Figure S15. Effects of Npx and IGF-1 mimetic peptide on inflammation and cholesterol efflux. (A) qRT-PCR analysis of TNFα and IL-1β in macrophage after indicated treatment, n=5. (B) Cholesterol efflux of peritoneal macrophage were assessed after treatment as indicated, n=5. Data are presented as mean ± SEM, ***P < 0.001, ****P < 0.0001 significantly different as indicated. ns: not significantly different.
